# Normal BOLD Response to a Step CO_2_ Stimulus After Correction for Partial Volume Averaging

**DOI:** 10.3389/fphys.2021.639360

**Published:** 2021-06-14

**Authors:** Julien Poublanc, Reema Shafi, Olivia Sobczyk, Kevin Sam, Daniel M. Mandell, Lakshmikumar Venkatraghavan, James Duffin, Joseph A. Fisher, David J. Mikulis

**Affiliations:** ^1^Joint Department of Medical Imaging and the Functional Neuroimaging Laboratory, University Health Network, Toronto, ON, Canada; ^2^Department of Anesthesia and Pain Management, University Health Network, Toronto, ON, Canada; ^3^Department of Radiology and Radiological Sciences, Johns Hopkins University, United States; ^4^Department of Physiology, University of Toronto, Toronto, ON, Canada; ^5^Institute of Medical Science, University of Toronto, Toronto, ON, Canada

**Keywords:** cerebrovascular reactivity, BOLD, CO_2_, speed of response, blood arrival time, healthy subjects, partial volume correction, vascular territories

## Abstract

Cerebrovascular reactivity (CVR) is defined as the change in cerebral blood flow induced by a change in a vasoactive stimulus. CVR using BOLD MRI in combination with changes in end-tidal CO_2_ is a very useful method for assessing vascular performance. In recent years, this technique has benefited from an advanced gas delivery method where end-tidal CO_2_ can be targeted, measured very precisely, and validated against arterial blood gas sampling (Ito et al., 2008). This has enabled more precise comparison of an individual patient against a normative atlas of healthy subjects. However, expected control ranges for CVR metrics have not been reported in the literature. In this work, we calculate and report the range of control values for the magnitude (mCVR), the steady state amplitude (ssCVR), and the speed (TAU) of the BOLD response to a standard step stimulus, as well as the time delay (TD) as observed in a cohort of 45 healthy controls. These CVR metrics maps were corrected for partial volume averaging for brain tissue types using a linear regression method to enable more accurate quantitation of CVR metrics. In brief, this method uses adjacent voxel CVR metrics in combination with their tissue composition to write the corresponding set of linear equations for estimating CVR metrics of gray matter (GM), white matter (WM), and cerebrospinal fluid (CSF). After partial volume correction, mCVR and ssCVR increase as expected in gray matter, respectively, by 25 and 19%, and decrease as expected in white matter by 33 and 13%. In contrast, TAU and TD decrease in gray matter by 33 and 13%. TAU increase in white matter by 24%, but TD surprisingly decreased by 9%. This correction enables more accurate voxel-wise tissue composition providing greater precision when reporting gray and white matter CVR values.

## Introduction

Cerebrovascular reactivity (CVR) is defined as the change in cerebral blood flow produced by a change in a vasoactive stimulus. Methods for assessing changes in blood flow include ultrasound (McDonnell et al., 2013), SPECT, CT (Chen et al., 2006), and MRI (Vesely et al., 2001). Commonly used vasoactive stimuli include inhaled CO_2_ (Vesely et al., 2001; Liu et al., 2019), acetazolamide infusion (Settakis et al., 2003), and lowering of blood pressure. One of the more common methods uses inhaled CO_2_ during BOLD MR imaging for measurement of CVR. An advance method for delivering quantitative vasodilatory stimuli (Slessarev et al., 2007) has enabled more accurate quantitation of vascular reactivity in healthy subjects and in patients with vascular diseases altering CVR. Despite this, the accuracy of the measurement also depends on post-processing methods. Standard CVR models assume a linear relationship of the BOLD response with the partial pressure of end-tidal CO_2_ (PETCO_2_) for calculating the magnitude of the response. However, the CVR response is more complex than just a scaled version of PETCO_2_. Multiple studies have addressed the problem of modeling CVR responses with development of other methods for taking into account the speed of the response (Poublanc et al., 2015), the time delay (Champagne et al., 2019), and competition from other voxels in the brain (Duffin et al., 2018).

Another limitation of CVR mapping is the low spatial resolution of the CVR images on 3T MRI. Voxel size can be as small as 2 mm when using a BOLD acquisition with advanced imaging techniques such as multi-band imaging. However, most common voxel size would range from 3 to 4 mm. Such voxels will contain different tissue constituents resulting in partial volume averaging effects. Partial volume effects (PVE) occur when the intensities from gray matter (GM), white matter (WM), and CSF are averaged together within a voxel. However, using the proportion of each tissue constituent within large voxels, it is possible to apply a correction in order to separate the intensities of each tissue constituent. This proportion is derived from tissue segmentation using a high-resolution anatomical image. Partial volume correction (PVC) is critical in patients with cortical atrophy since they are more likely to have fewer pure voxels that contain only one tissue constituent compared to normal individuals. PVC has previously been applied in PET imaging (Rousset et al., 2007), ASL MRI (Asllani et al., 2008; Petr et al., 2011, 2013; Binnewijzend et al., 2013), and spectroscopic imaging (Lu et al., 2007; Erlandsson et al., 2012; Bhogal et al., 2020), but not to CVR imaging. Different methods exist to correct for PVE. One of the most straightforward methods is to mask out voxels with tissue fraction below a given threshold. In a study comparing blood flow between young and elderly subjects, voxels with less than 80% gray matter were disregarded (Asllani et al., 2009). However, for a voxel-wise correction, more advanced methods exist such as a Bayesian (Chappell et al., 2011) or a linear regression methods (Asllani et al., 2008). The linear regression method, first introduced by Asllani et al. (2008), is more common and has been used frequently to correct ASL maps.

In this study, we introduce new techniques to further refine the temporal and spatial analyses of CVR images. First, the standard CVR model is compared to the convolution model using the speed of CVR response metric introduced in a previous study. Second, a new model is proposed that utilizes the existing speed metric to measure a time delay (TD). Lastly, a linear regression is applied to correct PVE. This method uses adjacent voxel CVR metrics in combination with their tissue composition to write the corresponding set of linear equations solving for CVR metrics derived from GM, WM, and CSF. This correction is applied to all CVR metrics and results are reported in the major vascular territories of 45 normal subjects before and after correction for PVE.

## Materials and Methods

### Subjects

This study was approved by the Research Ethics Board of the University Health Network and conformed to the Declaration of Helsinki. Written informed consent was obtained in all 45 healthy volunteers (20 women). The age range included 20 individuals between 20 and 30 years of age, 20 between 31 and 60 years of age, and 5 over 60 years old. Mean age for all subjects was 39.2 ± 16.5 years. Subjects were in good health, non-smokers, and taking no medication.

### Vasodilatory Stimulus

Cerebrovascular reactivity was assessed by measuring the change in BOLD signal to a standardized change in end-tidal partial pressure of carbon dioxide (PETCO_2_) as the vasodilatory stimulus. PETCO_2_ and end-tidal partial pressure of oxygen (PETO_2_) were targeted independently of each other and of the subjects’ minute ventilation and breathing pattern, by using an automated gas blender and sequential gas delivery breathing circuit (Slessarev et al., 2007) (RespirAct; Thornhill Medical, Toronto, ON, Canada). Targeted PETCO_2_ and PETO_2_ was achieved within 1–2 breaths by administering blends of gasses according to a previously described algorithms (Slessarev et al., 2007). The targeting sequence used in this study was as follows: (1) maintaining resting PETCO_2_ for 2 min, (2) a step increase of 10 mmHg PETCO_2_ above the resting level for 2 min, and (3) a step down decrease in PETCO_2_ for 2 min back to the resting PETCO_2_ level.

### Image Acquisition

MR imaging was performed on a 3T system (Signa HDX GE Healthcare, Milwaukee, WI, United States) using an 8-channel phased array head coil. A BOLD acquisition was applied with the following parameters: gradient-echo, echo-planar sequence, TE/TR = 30/2,400 ms, 22.4 × 22.4 cm FOV, 64 × 64 matrix, 39 slices, 3.5 mm isotropic voxels, no interslice gap, 338 temporal frames, and 85°flip angle. For anatomical reference, a 3D T1-weighted inversion recovery fast-spoiled gradient-recalled sequence was used with the following parameters: TI/TR/TE, 450/8/3 ms; matrix size, 256 × 256; FOV, 22 × 22 cm; slice sickness, 1 mm; and flip angle, 15°.

### Image Analysis

MRI and PETCO_2_ data were transferred to an independent workstation and preprocessed using AFNI (Cox, 1996; Cox and Hyde, 1997) and Matlab 2015a. Functional BOLD images were volume registered, slice-time corrected, and co-registered to the anatomical T_1_-weighted scan.

### Standard CVR Model

**FIGURE 1:**
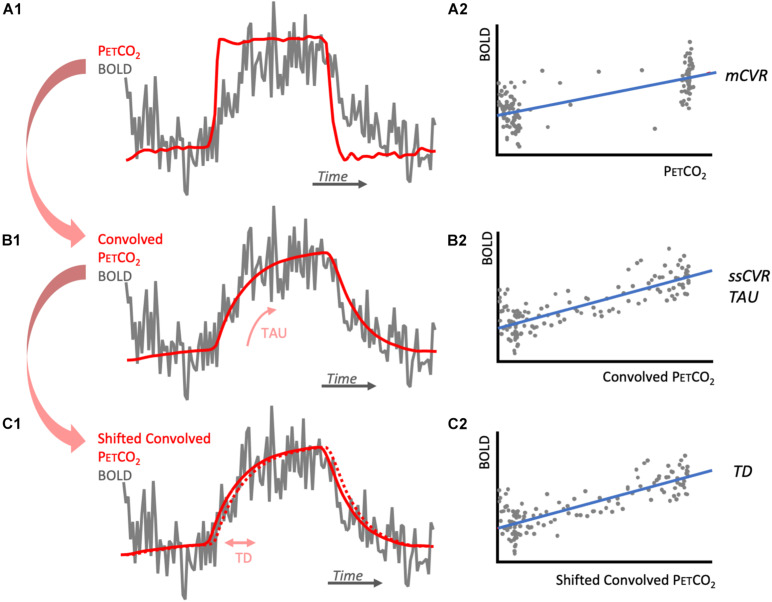
Diagrams illustrating CVR metrics calculations. **(A1)** represents one voxel BOLD response (gray) to the step PETCO_2_ (red). BOLD signal is regressed against PETCO_2_ to obtain its slope, mCVR **(A2)**. The PETCO_2_ is then convolved with an exponential function of time characteristic TAU that is chosen to maximize the correlation with the BOLD response **(B1)**. BOLD signal is then regressed against the convolved PETCO_2_ to obtained its slope, ssCVR **(B2)**. The convolved PETCO_2_ is then shifted to obtain maximum correlation with the convolved PETCO_2_. This shift corresponds to TD **(C1)** and the adjusted regression is represented in **(C2)**. Notes that for each of the regressions **(A2,B2,C2)**, BOLD signal drift is added to this model as a linear trend. For simplification, it is not represented here.

PETCO_2_ data were re-sampled and time-shifted to the point of coincidence between the rapid changes in PETCO_2_ and BOLD signal using Matlab. Magnitude CVR (mCVR) was calculated on a voxel-by-voxel basis from the slope of a linear least-squares fit of the BOLD signal data series to the PETCO_2_ values obtained from the RespirAct. For this regression, the rise of PETCO_2_ and brain average BOLD signal were matched (Figures 1A1,A2). CVR is expressed as the percent change in BOLD signal per mmHg change in PETCO_2_. BOLD signal drift was controlled by adding a linear trend in time to the model. The mathematical expression of this model is as follow:

(1) $$S\left(t\right)=mCVR\times P\text{ET}CO_{2}\left(t\right)+\alpha \times t+b\left(t\right)+\varepsilon \left(t\right)$$

Variables *α*×*t* account for linear BOLD signal drift; b(t) is a constant baseline over time; ε(t) is the residual time series. mCVR is the slope of the regression of the BOLD signal for all the acquired datapoints in each voxel during the stimulus.

### Convolution CVR Model

BOLD response was modeled as the PETCO_2_ convolved with an exponential decay function exp(−*t*/TAU), where *t* is time and TAU is the time constant of the vascular response. TAU was allowed to vary from 2 to 100 s in 2 s increments. TAU derived from the convolved PETCO_2_ with the highest Pearson correlation coefficient with the BOLD response was taken as a measure of the speed of response (TAU in Figure 1B1). Steady state CVR (ssCVR) was calculated as the slope of the regression between the BOLD response and the convolved PETCO_2_ waveform that provided the highest correlation (Figure 1B2). TAU is expressed in seconds and ssCVR is expressed as percent MR signal change per mmHg of PETCO_2_.

The mathematical expression of this model is as follows:

(2) $$\begin{array}{c} S\left(t\right)=ssCVR\times \left\{P\text{ET}CO_{2}\left(t\right)⊗\exp\left(-t/\mathrm{TAU}\right)/C\right\}\\ +\alpha \times t+b\left(t\right)+\varepsilon \left(t\right) \end{array}$$

where *α* × *t*, b(t), and ε(t) are the same measures as in (1).

$\text{C}=\int_{0}^{5\times TAU}\varepsilon^{-t/TAU}dt$ is a normalization constant. {*P*ET*CO*_2_(*t*)⊗exp(−t/TAU)/C} is the convolved PETCO_2_ later used in the text and figures (Figures 1B1,B2). Note that ssCVR can be thought of as CVR corrected for the speed of the response TAU. This method is explained in more detail elsewhere (Poublanc et al., 2015).

### Time Delay (TD)

The convolved PETCO_2_ previously calculated was first shifted 2 s earlier in time to ensure it precedes BOLD signal for all voxels for the brain. It was then shifted to the point of maximum correlation with BOLD response, on a voxel-wise basis (Figures 1C1,C2). This shift represents TD and was allowed to vary between 0 and 5 s, in increments of 0.2 s. The mathematical expression for this is:

(3) $$\begin{array}{c} S\left(t\right)=ssCVR\times Convolved\_P\text{ET}CO_{2}\left(t-TD\right)\\ +\alpha \times t+b\left(t\right)+\varepsilon \left(t\right) \end{array}$$

where *α* × *t*, b(t), and ε(t) are the same measures as in (1).

Convolved_ PETCO_2_ is the modified PETCO_2_ found in (2). Due to the small shift in the convolved PETCO_2_, ssCVR value can vary slightly from the value found in (2).

### Spatial Normalization and Segmentation

Statistical Parametric Mapping (SPM) 8 software was used to segment and spatially normalize the high resolution T1-weighted anatomical acquisition (Ashburner and Friston, 2005). The segmentation provides probability maps of gray matter (GM), white matter (WM), and cerebrospinal fluid (CSF). GM, WM, and CSF masks were generated for each patient by thresholding the corresponding probability maps at 0.7.

### Nearest Neighbor Interpolation (NN)

All CVR metrics maps were re-sampled to the T1-weighted resolution using NN interpolation.

### Partial Volume Correction (PVC)

**FIGURE 2:**
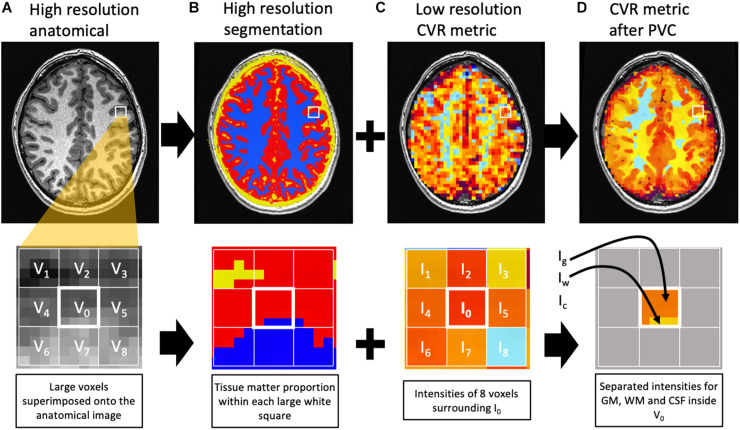
Diagram representing the partial volume correction algorithm. The top row represents one slice of the brain, with a closeup on the second row. The high-resolution anatomical image **(A)** is used for segmentation **(B)** of GM (red), WM (blue), and CSF (yellow). Within the large voxels of the low-resolution image [white grid in **(C)**], the proportion of each tissue matter can be calculated [**(B)**, second row]. For example, voxel V_0_ contains 81% gray matter (g_0_), 19% white matter (w_0_), and 0% CSF (c_0_). The intensity I_0_ in V_0_ is an average of the intensities of GM (I_*g*_), WM (I_*w*_), and CSF (I_*c*_) weighted by their respective matter proportion. The corresponding equation would be I_0_ = g_0_ I_*g*_ + w_0_ I_*w*_ + c_0_ I_*c*_. Under the assumption that each matter intensity (I_*g*_, I_*w*_, and I_*c*_) is constant within a group of voxels surrounding V_0_, 8 similar equations can be written for V_1_, V_2_, …, V_8_ to solve for I_*g*_, I_*w*_, and I_*c*_ inside V_0_. I_*g*_, I_*w*_, and I_*c*_ are then assigned to the corresponding tissue matter inside V_0_ **(D)**. Notes that voxels surrounding the central one but outside the BOLD mask are excluded from the regression. In this example, the solution I_*c*_ is not assigned since there is no CSF inside V_0_. For this example, only 8 voxels surround V_0_. In the actual image analysis, the surrounding voxels were all voxels within a 9 mm radius.

The proportion of GM, WM, and CSF within the large BOLD voxels (3.5 × 3.5 × 3.5 mm) in the functional BOLD imaging protocol differs between subjects. Since normal cortical gray matter is approximately 2.5 mm in thickness, all 3.5 mm isotropic voxels over cortical structures (except for a few voxels where gyral surfaces are in contact with each other across a sulcus) will contain less than 100% gray matter (Figure 2). This can introduce an underestimation bias in the voxel content of gray matter, particularly in the setting of cortical atrophy. The following method was applied to correct for this volume averaging. Let’s consider the BOLD acquisition voxel V_0_ with signal intensity I_0_. Note that I_0_ could represent mCVR, TAU, ssCVR, or TD. I_0_ is equal to the weighted average of the intensity of GM (I_*g*_), WM (I_*w*_), and CSF (I_*c*_) that corresponds to I_0_ = g_0_ I_*g*_ + w_0_ I_*w*_ + c_0_ I_*c*,_ where g_0_, w_0_, and c_0_ are the proportion of GM, WM, and CSF inside V_0_. This equation cannot be solved by itself since there are 3 unknowns (I_*g*_, I_*w*_, and I_*c*_). In order to solve it, surrounding voxels are needed. Under the assumption that the intensity of each tissue constituent is constant within the surrounding voxels, a multiple linear regression model can be used to solve for I_*g*_, I_*w*_, and I_*c*_. In this analysis surrounding voxels were all voxels within a 9 mm radius. This yields 81 voxels including V_0_ corresponding to 81 equations: I_*n*_ = g_*n*_ I_*g*_ + w_*n*_ I_*w*_ + c_*n*_ I_*c*_ + ε_*n*_ where n = 0,1,… 80.

For each BOLD voxel n, the quantities g_*n*_, w_*n*_, and c_*n*_ represent the proportion of GM, WM, and CSF, respectively, in the BOLD voxel. ε_*n*_ represents the error. The unknown quantities I_*g*_, I_*w*_, and I_*c*_ are the intensity of GM, WM, and CSF in the BOLD voxels. The proportions of the 3 tissue classes within the BOLD voxel are obtained from the high resolution T1-weighted segmentation described earlier that has 1 mm isotropic voxels. After solving the linear equations using linear regression, I_*g*_, I_*w*_, and I_*c*_ are assigned to the correct tissue class inside V_0_. The same process is then repeated for all voxels of the brain. Note that voxels inside the 9 mm radius but outside the BOLD mask are excluded from the regression. This method was implemented in a script using multiple AFNI functions and is similar to previous work (Asllani et al., 2008; Petr et al., 2013; Ahlgren et al., 2014). Figure 2 illustrates the process. 3dTfitter from AFNI was used for the linear regression. The regression was applied by minimizing the sum of absolute errors, rather than the sum of square since it is less sensitive to outliers. The correction was repeated for each CVR metric. In addition, mCVR was corrected for partial volume averaging using different radii (5, 7, 9, and 11 mm) to illustrate the change in intensity with radius size.

### Vascular Territories Template (VTT)

A template of vascular territories, available online and constructed and reported in a 2019 study (Schirmer et al., 2019), was used in this analysis. Those territories included the cerebellum (CER), anterior cerebral arteries (ACA), middle cerebral artery (MCA), and the posterior cerebral artery (PCA) territories. The inverse transformation matrix previously calculated for each subject was used to transform the VTT back to subject original space, rather than transforming the functional maps to MNI. Indeed, warping the functional maps to MNI would introduce re-sampling, leading to a sub-optimal partial volume correction.

### CVR Metrics Averages

The average of CVR metrics (mCVR, ssCVR, TAU, and TD) for the PVC and the NN interpolation maps were calculated within GM of each vascular territory, as well as within WM. We attempted to exclude some of the sinovenous structures from the analysis by removing all voxels with mCVR above 0.7%/mmHg. In addition, voxels with negative ssCVR were removed from TAU averages. TAU in those voxels does not represent the speed of vasodilation since those voxels are likely driven by a steal phenomenon from stronger CVR responses in adjacent neighbors. In normal controls, there are only a small number of voxels with this negative response.

### Model Selection

Bayesian Information Criterion (BIC) was calculated for the standard CVR model, as well as for the convolution model, in order to compare them. The model with the lowest BIC corresponds to the best model.

BIC=nln(SSE/n)+(p+1)ln(n)

SSE is the residual sum of squares, n is the number of time points, and p is the number of parameters used in the model. The second term in the BIC expression introduces a penalty when adding more parameters to the model. For the standard model (Eq. 1), there are 2 parameters (mCVR and), thus *p* = 2, and for the convolution model (Eq. 2), there are 3 parameters (ssCVR, TAU, and), thus *p* = 3. BIC was averaged within gray and white matter separately for each model.

## Results

**FIGURE 3:**
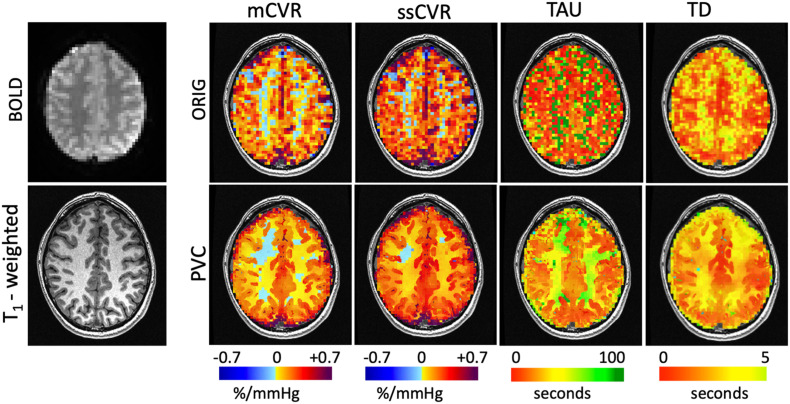
Maps of CVR metrics (mCVR, ssCVR, TAU, and TD) for one subject represented using the original resolution (ORIG) in the first row and processed using the partial volume correction (PVC) in the second row. The raw BOLD image and the high resolution T_1_ weighted image are shown in the first column. Using the matter segmentation from the T_1_-weighted high resolution (1 mm isotropic) scan, the original CVR metrics maps of 3.5 mm isotropic resolution (ORIG) were corrected for partial volume effect and mapped onto the 1 mm isotropic grid.

The BOLD response in one voxel of one subject is shown in the diagram of Figure 1. This clearly shows that the rise in BOLD signal following a step hypercapnia stimulus is not instantaneous but rather follows an exponential function. An example of mCVR, ssCVR, TAU, and TD maps generated in one subject are shown in Figure 3 in original space and original resolution (ORIG). The same maps were then resampled onto the anatomical space using nearest neighbor mode (NN) for comparison with the partial volume corrected maps (PVC).

Using the nearest neighbor resampled maps, average GM and WM were 0.17 ± 0.03 and 0.09 ± 0.02%/mmHg for mCVR, 0.25 ± 0.05 and 0.15 ± 0.03%/mmHg for ssCVR, 24.7 ± 7.6 and 39.0 ± 6.1s for TAU, and 2.3 ± 0.5 and 3.0 ± 0.5s for TD. Using partial volume corrected maps, average GM and WM were 0.22 ± 0.04 and 0.06 ± 0.02%/mmHg for mCVR, 0.30 ± 0.04 and 0.13 ± 0.03%/mmHg for ssCVR, 23.5 ± 8.1 and 48.7 ± 11.2 s for TAU, and 2.2 ± 0.8 and 2.8 ± 0.9 s for TD.

**FIGURE 4:**
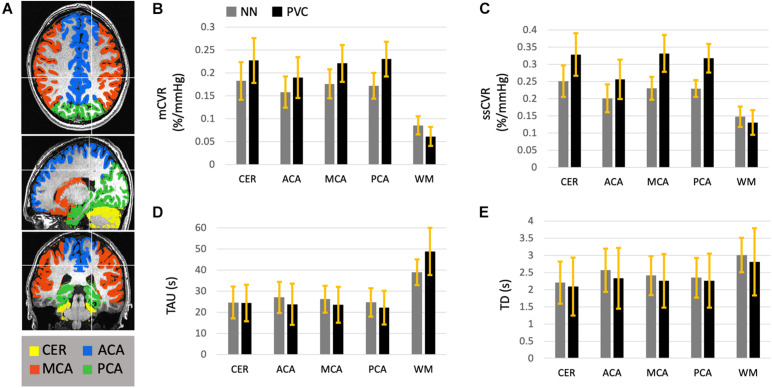
This diagram represents the average mCVR **(B)**, ssCVR **(C)**, TAU **(D)**, and TD **(E)** over five gray matter ROIs and over 45 normal controls. The ROIs **(A)** consist of gray matter in the cerebellum (CER), the anterior (ACA), middle (MCA), and posterior (PCA) cerebral artery territories, as well as all hemispheric white matter (WM). This diagram shows the results after CVR metrics maps were resampled to the high resolution anatomical scan using nearest neighbor (NN, in gray), as well as corrected for partial volume effect (PVC in black). The standard deviations are represented with the yellow bars.

After partial volume correction, mCVR and ssCVR increase in gray matter, respectively, by 25 and 19%, and decrease in white matter by 33 and 13%. In contrast, TAU and TD decrease in gray matter by 33 and 13%. TAU increases in white matter by 24% and TD surprisingly decreases by 9%. Overall, this indicates that PVC effectively draws gray and white matter measures toward their true values. Measurements for gray matter of each separate vascular territory follows the same tendency as for the whole gray matter. Those results are combined in Table 1 and Figure 4. In addition, the results performed on mCVR at different radii sizes were the following: the average GM for NN, PVC with the 5, 7, 9, and 11 mm radii were, respectively, 0.17 ± 0.03, 0.19 ± 0.04, 0.20 ± 0.03, 0.22 ± 0.04 %/mmHg, and 0.22 ± 0.04 %/mmHg. The average WM for NN, PVC with the 5, 7, 9, and 11 mm radii were, respectively, 0.09 ± 0.02, 0.05 ± 0.02, 0.05 ± 0.02, 0.06 ± 0.02 %/mmHg, 0.07 ± 0.02 %/mmHg.

**TABLE 1:** Average CVR metrics (mCVR, ssCVR, TAU, BAT) within white matter and gray matter vascular territories (Cerebellum, ACA, MCA, and PCA), before and after partial volume correction, in normal healthy subjects.

		mCVR (%/mmHg)	ssCVR (%/mmHg)	TAU (s)	TD (s)
Cerebellum (Gray matter)	No corr. PVC	0.18 ± 0.04 0.23 ± 0.05	0.25 ± 0.05 0.33 ± 0.06	24.7 ± 7.6 24.4 ± 8.7	2.2 ± 0.6 2.1 ± 0.8
ACA (Gray matter)	No corr. PVC	0.16 ± 0.03 0.19 ± 0.04	0.20 ± 0.04 0.26 ± 0.07	27.1 ± 7.4 23.8 ± 9.8	2.6 ± 0.6 2.3 ± 0.9
MCA (Gray matter)	No corr. PVC	0.18 ± 0.03 0.22 ± 0.04	0.23 ± 0.03 0.33 ± 0.05	26.2 ± 6.4 23.5 ± 8.4	2.4 ± 0.6 2.3 ± 0.8
PCA (Gray matter)	No corr. PVC	0.19 ± 0.03 0.23 ± 0.04	0.23 ± 0.02 0.32 ± 0.04	24.7 ± 6.7 22.2 ± 7.9	2.3 ± 0.6 2.3 ± 0.8
White matter	No corr. PVC	0.09 ± 0.02 0.06 ± 0.02	0.15 ± 0.03 0.13 ± 0.04	39.0 ± 6.1 48.7 ± 11.2	3.0 ± 0.5 2.8 ± 0.9

Average BIC over gray matter was 76.6 ± 58.7 and 61.2 ± 63.8 for the standard and the convolution model, respectively. For white matter, average BIC was 58.5.6 ± 50.0 and 48.0 ± 53.1 for the standard and the convolution model, respectively. BIC values for all individual subjects were smaller for the convolution model than for the standard model (*p* < 0.001).

## Discussion

In this study, we provide an algorithm that improves the accuracy of CVR measurements in the brain by reducing volume averaging artifacts arising from mixtures of these three tissue classes within the relatively large voxels used in BOLD MRI. This becomes particularly important in aging individuals and in patients with disease related cerebral atrophy. The degree to which averaging of the different signal intensities of GM, WM, and CFS is controlled reduces misclassification and dependence on voxel size. This correction is particularly important when comparing patients with cerebral atrophy or white matter hyper intensities against a healthy control population (Thomas et al., 2011). The model for this correction assumes uniform intensity of each of the GM, WM, and CSF signal intensities within a small region. However, the smaller the radius, the more unstable the linear regression will be. On the other hand, the assumption of uniform intensity is likely to be false over larger radii. Our results shows that the average GM increases with radius sizes before plateauing at a radius size of 9 mm. The average WM decreases from NN resampling to PVC at 5 mm radius and then increases slightly with radius size. We have chosen a 9 mm radius; however, a simulation would have to be performed to clarify the optimum radius for obtaining the highest precision. In this study, the BOLD signal from CSF was not assumed to be null as it often is when correcting ASL images for partial volume effect. CSF signal was incorporated in the linear regression since large BOLD signal in surface veins can extend to the surrounding CSF.

Since this technique requires high resolution T1-weighted images for segmentation, co-registration with the CVR is an important step. Our T1-weighted images were co-registered to the CVR raw images with a rigid body registration and visually inspected. The correction was applied on the CVR metric maps in original space. Vascular territories were transformed from MNI space to original space. This is an important point since applying the correction to the maps in MNI space or transforming the corrected maps in MNI space would have introduced additional spatial re-sampling and smoothing, diverging from the purpose of partial volume correction. The algorithm requires neighbor information that provides a more precise classification accuracy for CVR. Further work is needed to test this algorithm on various disease groups using similar testing parameters, to assess consistency, and to evaluate its impact on improving classification accuracy.

Improving accuracy may be possible by examining the point spread function associated with signal bleeding from the low resolution acquisition. This PSF can then be applied to the anatomical image before segmentation for a better understanding of the low resolution contribution of the different intensities from the three tissue classes. This adjustment can be used before the regression to correct for partial volume effect as shown in spectroscopic imaging in a recent study (Lu et al., 2007). However, considering the size of the BOLD imaging voxels (3.5 mm isotropic) compared to the spectroscopic voxels (5 × 5 × 10 mm), the contribution is expected to have a smaller effect, but it is worthwhile to examine to further improve the current implementation.

Note that the partial volume correction was applied on the parametric images as opposed to the raw BOLD signal. Both approaches should be equivalent when the model is linear such as the one used to generate mCVR. However, we might observe more discrepancy when the model is non-linear such as the one used to extract TAU and ssCVR. Although applying this correction on the raw BOLD signal is processing intensive, this comparison might need to be investigated in the future.

Adjusting for the speed of the response in the model also improves the fit significantly. Indeed, taking the number of parameters used in each model, the BIC for the convolution model is smaller than for the standard model. Although the convolved exponential model is able to fit BOLD responses from healthy as well as diseased vasculature, the redistribution of blood flow in the presence of steal physiology in the setting of steno-occlusive cerebrovascular disease remains problematic. The negative TAU under these conditions no longer accurately indexes the degree of vascular disease. In the setting of steal physiology negative TAU can be paradoxically short. Under these pathological states, the CO_2_ stimulus first arrives in the circulation of tissue with intact vasodilatory responses. This circulation lowers its resistance to flow followed by rapid redirection of blood flow away from tissue that cannot change its flow resistance as it is already markedly dilated in attempting to maintain resting blood flow at normal levels. Other methods have used cross correlation time-shift to extract a measure of time delay. However, a cross-correlation technique cannot separate the blood arrival time component from the response time component. In fact, the use of just an amplitude and a shift poorly models the step BOLD response since the main time delay component appears as a slow rise of the response rather than a shift in the response. The solution we provide here is to introduce a time shift parameter (TD) to the convolution model. Note that TD attempts to measure blood arrival time. However, as opposed to ASL and DSC imaging, cerebral blood flow is altered during CO_2_ manipulations. Under those circumstances, blood flow will be redistributed, and true blood arrival time might be altered. Therefore, this parameter was not named blood arrival time but time delay. TAU contributes to a much larger component than TD in the model fit. Time delay differences across the brain should not exceed more than 3–4 s (Poublanc et al., 2013), only marginally improving the measurement particularly with a 120 s hypercapnia stimulus. In addition, the solution is limited by the protocol as we only applied a single hypercapnic step, and temporal resolution is low with BOLD images acquired at TR of 2.4 s. The protocol used is better suited to measure TAU rather than TD. Future work may explore the use of multiple short CO_2_ steps to improve the performance of TD measurement. In addition, blood arrival time could be altered with increasing hypercapnia since a rise in CO_2_ induces an increase in the speed of blood flow. Nevertheless, variations in TD across diseased states relative to healthy control participants allows for group and regional comparisons such as brain matter type, hemispheric asymmetries, and variability across vascular territories. In particular, we propose that this added metric may hold greater promise for assessments pertaining to vascular aging and its correlation to cognitive reserve.

Our rationale for using a standard step PETCO_2_ protocol has been guided by the fact that our method for increasing arterial PETCO_2_ 10 mmHg above resting levels can be achieved in one breath. The sharp step rise and fall in CO_2_ enabled clear observation of the BOLD response that led to identification of the exponential rise in signal. Various research groups have also utilized a more attenuated step protocol across numerous published studies (Goode et al., 2009; Anazodo et al., 2016; Leung et al., 2016; Moreton et al., 2016). Over time, the sharp step protocol has, in part, become a standard stimulus largely due to its value in measuring the speed of vasodilation while reducing scan time.

Finally, this report provides reference values of the derived CVR metrics in the vascular territories of the healthy human brain. One limitation is the heterogeneity of age within the study group. However, we have previously shown that, based on our published methods, there are no significant differences in the speed and magnitude of CVR and importantly these values do not differ in GM regions amongst various age groups across the healthy aging continuum, i.e., 18–54 (McKetton et al., 2018). We recognize that there are differences by age documented in some studies (Bhogal et al., 2016; Peng et al., 2018) in the literature. However, the ability to rapidly control and implement precise known changes in arterial CO_2_ levels as used in this study attests to the accuracy of the data and the conclusions. However, we note that the sample of participants in our older age group is limited and more investigation is needed to determine the effects of healthy aging over the seventh decade of life.

## Data Availability Statement

The original contributions presented in the study are included in the article/supplementary material, further inquiries can be directed to the corresponding author/s.

## Ethics Statement

The studies involving human participants were reviewed and approved by the University Health Network. The patients/participants provided their written informed consent to participate in this study. The patients/participants provided their written informed consent to participate in this study.

## Author Contributions

JP was involved in the development of the scripts development and image analysis. All authors participated in the design and conceptualization of the study, as well as the feedback and writing process following the initial drafting of the manuscript, contributed to the article, and approved the submitted version.

## Conflict of Interest

JF and DJM contributed to the development of the automated end-tidal targeting device, RespirAct^TM^ (Thornhill Medical Inc.) used in this study and have equity in the company. The remaining authors declare that the research was conducted in the absence of any commercial or financial relationships that could be construed as a potential conflict of interest.

## References

[B1] Ahlgren A. Wirestam R. Petersen E. T. Ståhlberg F. Knutsson L. (2014). Partial volume correction of brain perfusion estimates using the inherent signal data of time-resolved arterial spin labeling. *NMR Biomed* 27 1112–1122. 10.1002/nbm.3164 25066601

[B2] Anazodo U. C. Shoemaker J. K. Suskin N. Ssali T. Wang D. J. St Lawrence K. S. (2016). Impaired cerebrovascular function in coronary artery disease patients and recovery following cardiac rehabilitation. *Front. Aging Neurosci.* 7:224. 10.3389/fnagi.2015.00224 26779011 PMC4700211

[B3] Ashburner J. Friston K. J. (2005). Unified segmentation. *Neuroimage* 26 839–851. 10.1016/j.neuroimage.2005.02.018 15955494

[B4] Asllani I. Borogovac A. Brown T. R. (2008). Regression algorithm correcting for partial volume effects in arterial spin labeling MRI. *Magn. Reson. Med.* 60 1362–1371. 10.1002/mrm.21670 18828149

[B5] Asllani I. Habeck C. Borogovac A. Brown T. R. Brickman A. M. Stern Y. (2009). Separating function from structure in perfusion imaging of the aging brain. *Hum. Brain Mapp.* 30 2927–2935. 10.1002/hbm.20719 19172645 PMC2733928

[B6] Bhogal A. A. Broeders T. A. A. Morsinkhof L. Edens M. Nassirpour S. Chang P. (2020). Lipid-suppressed and tissue-fraction corrected metabolic distributions in human central brain structures using 2D 1 H magnetic resonance spectroscopic imaging at 7 T. *Brain Behav.* 10:e01852. 10.1002/brb3.1852 33216472 PMC7749561

[B7] Bhogal A. A. De Vis J. B. Siero J. C. W. Petersen E. T. Luijten P. R. Hendrikse J. (2016). The BOLD cerebrovascular reactivity response to progressive hypercapnia in young and elderly. *Neuroimage* 139 94–102. 10.1016/j.neuroimage.2016.06.010 27291492

[B8] Binnewijzend M. A. A. Kuijer J. P. A. Benedictus M. R. van der Flier W. M. Wink A. M. Wattjes M. P. (2013). Cerebral blood flow measured with 3D pseudocontinuous arterial spin-labeling MR imaging in alzheimer disease and mild cognitive impairment: a marker for disease severity. *Radiology* 267 221–230. 10.1148/radiol.12120928 23238159

[B9] Champagne A. A. Bhogal A. A. Coverdale N. S. Mark C. I. Cook D. J. (2019). A novel perspective to calibrate temporal delays in cerebrovascular reactivity using hypercapnic and hyperoxic respiratory challenges. *Neuroimage* 187 154–165. 10.1016/j.neuroimage.2017.11.044 29217405

[B10] Chappell M. A. Groves A. R. MacIntosh B. J. Donahue M. J. Jezzard P. Woolrich M. W. (2011). Partial volume correction of multiple inversion time arterial spin labeling MRI data. *Magn. Reson. Med.* 65 1173–1183. 10.1002/mrm.22641 21337417

[B11] Chen A. Shyr M. H. Chen T. Y. Lai H. Y. Lin C. C. Yen P. S. (2006). Dynamic CT perfusion imaging with acetazolamide challenge for evaluation of patients with unilateral cerebrovascular steno-occlusive disease. *Am. J. Neuroradiol.* 27 1876–1881. 17032859 PMC7977914

[B12] Cox R. W. A. F. N. I. (1996). Software for analysis and visualization of functional magnetic resonance neuroimages. *Comput. Biomed. Res.* 29 162–173. 10.1006/cbmr.1996.0014 8812068

[B13] Cox R. W. Hyde J. S. (1997). Software tools for analysis and visualization of fMRI data. *NMR Biomed* 10 171–178. 10.1002/(SICI)1099-1492(199706/08)10:4/5<171::AID-NBM453<3.0.CO;2-L 9430344

[B14] Duffin J. Sobczyk O. McKetton L. Crawley A. Poublanc J. Venkatraghavan L. (2018). Cerebrovascular resistance: the basis of cerebrovascular reactivity. *Front. Neurosci.* 12:409. 10.3389/fnins.2018.00409 29973862 PMC6020782

[B15] Erlandsson K. Buvat I. Pretorius P. H. Thomas B. A. Hutton B. F. (2012). A review of partial volume correction techniques for emission tomography and their applications in neurology, cardiology and oncology. *Phys. Med. Biol.* 57 R119–R159. 10.1088/0031-9155/57/21/R119 23073343

[B16] Goode S. D. Krishan S. Alexakis C. Mahajan R. Auer D. P. (2009). Precision of cerebrovascular reactivity assessment with use of different quantification methods for hypercapnia functional MR imaging. *Am. J. Neuroradiol.* 30 972–977. 10.3174/ajnr.A1496 19435945 PMC7051672

[B17] Ito S. Mardimae A. Han J. Duffin J. Wells G. Fedorko L. (2008). Non-invasive prospective targeting of arterial PCO2 in subjects at rest. *J. Physiol.* 586 3675–3682. 10.1113/jphysiol.2008.154716 18565992 PMC2538829

[B18] Leung J. Duffin J. Fisher J. A. Kassner A. (2016). MRI-based cerebrovascular reactivity using transfer function analysis reveals temporal group differences between patients with sickle cell disease and healthy controls. *NeuroImage Clin.* 12 624–630. 10.1016/j.nicl.2016.09.009 27722086 PMC5048082

[B19] Liu P. De Vis J. B. Lu H. (2019). Cerebrovascular reactivity (CVR) MRI with CO2 challenge: a technical review. *Neuroimage* 187 104–115. 10.1016/j.neuroimage.2018.03.047 29574034 PMC6150860

[B20] Lu Y. Wu D. Magnotta V. A. (2007). “Partial volume correction of magnetic resonance spectroscopic imaging,” in *Medical Imaging: Image Processing*, eds Pluim J. P. W. Reinhardt J. M. (SPIE), 651243.

[B21] McDonnell M. N. Berry N. M. Cutting M. A. Keage H. A. Buckley J. D. Howe P. R. (2013). Transcranial doppler ultrasound to assess cerebrovascular reactivity: reliability, reproducibility and effect of posture. *Peer J.* 1:e65. 10.7717/peerj.65 23646284 PMC3642776

[B22] McKetton L. Sobczyk O. Duffin J. Poublanc J. Sam K. Crawley A. P. (2018). The aging brain and cerebrovascular reactivity. *Neuroimage* 181 132–141. 10.1016/j.neuroimage.2018.07.007 29981482

[B23] Moreton F. C. Dani K. A. Goutcher C. O’Hare K. Muir K. W. (2016). Respiratory challenge MRI: practical aspects. *NeuroImage Clin.* 11 667–677. 10.1016/j.nicl.2016.05.003 27330967 PMC4901170

[B24] Peng S. L. Chen X. Li Y. Rodrigue K. M. Park D. C. Lu H. (2018). Age-related changes in cerebrovascular reactivity and their relationship to cognition: a four-year longitudinal study. *Neuroimage* 174 257–262. 10.1016/j.neuroimage.2018.03.033 29567504 PMC5949266

[B25] Petr J. Schramm G. Hofheinz F. Langner J. van den Hoff J. (2013). Partial volume correction in arterial spin labeling using a look-locker sequence. *Magn. Reson. Med* 70 1535–1543. 10.1002/mrm.24601 23280559

[B26] Petr J. Schramm G. Hofheinz F. Langner J. van den Hoff J. (2011). Partial volume correction in arterial spin labeling using a look-locker sequence. *Magn. Reson. Med.* 65 1173–1183. 23280559 10.1002/mrm.24601

[B27] Poublanc J. Crawley A. P. Sobczyk O. Montandon G. Sam K. Mandell D. M. (2015). Measuring cerebrovascular reactivity: the dynamic response to a step hypercapnic stimulus. *J. Cereb. Blood Flow. Metab.* 35 1746–1756. 10.1038/jcbfm.2015.114 26126862 PMC4635229

[B28] Poublanc J. Han J. S. Mandell D. M. Conklin J. Stainsby J. A. Fisher J. A. (2013). Vascular steal explains early paradoxical blood oxygen level-dependent cerebrovascular response in brain regions with delayed arterial transit times. *Cerebrovasc Dis. Extra.* 3 55–64. 10.1159/000348841 24052795 PMC3776710

[B29] Rousset O. Rahmim A. Alavi A. Zaidi H. (2007). Partial volume correction strategies in PET. *PET Clin.* 2 235–249. 10.1016/j.cpet.2007.10.005 27157875

[B30] Schirmer M. D. Giese A.-K. Fotiadis P. Etherton M. R. Cloonan L. Viswanathan A. (2019). Spatial signature of white matter hyperintensities in stroke patients. *Front. Neurol.* 10:208. 10.3389/fneur.2019.00208 30941083 PMC6433778

[B31] Settakis G. Molnár C. Kerényi L. Kollár J. Legemate D. Csiba L. (2003). Acetazolamide as a vasodilatory stimulus in cerebrovascular diseases and in conditions affecting the cerebral vasculature. *Eur. J. Neurol.* 10 609–620. 10.1046/j.1468-1331.2003.00675.x 14641504

[B32] Slessarev M. Han J. Mardimae A. Prisman E. Preiss D. Volgyesi G. (2007). Prospective targeting and control of end-tidal CO2 and O2 concentrations. *J. Physiol.* 581(Pt. 3) 1207–1219. 10.1113/jphysiol.2007.129395 17446225 PMC2170842

[B33] Thomas B. A. Erlandsson K. Modat M. Thurfjell L. Vandenberghe R. Ourselin S. (2011). The importance of appropriate partial volume correction for PET quantification in Alzheimer’s disease. *Eur. J. Nucl. Med. Mol. Imaging* 38 1104–1119. 10.1007/s00259-011-1745-9 21336694

[B34] Vesely A. Sasano H. Volgyesi G. Somogyi R. Tesler J. Fedorko L. (2001). MRI mapping of cerebrovascular reactivity using square wave changes in end-tidal PCO2. *Magn. Reson. Med.* 45 1011–1013. 10.1002/mrm.1134 11378878

